# Exploring Iranian sentiments on the Paris Agreement: Insights from Twitter

**DOI:** 10.1016/j.heliyon.2025.e42716

**Published:** 2025-02-14

**Authors:** Faeze Atefinia, Seyed Reza Mirnezami

**Affiliations:** The Research Institute for Science, Technology, and Industrial Policy, Sharif University of Technology, Azadi St, Tehran, 11365-8639, Iran

**Keywords:** Paris agreement, Iran, Public opinion, Twitter, Developing countries

## Abstract

Iran remains one of the few nations not fully committed to the Paris Agreement and public opinion regarding implementation of policies serves a significant role. This study examines the general sentiment of Iranian Twitter users towards the Paris Agreement. A total of 25,386 tweets were collected and analyzed, revealing that discussions around the Paris Agreement predominantly centred on terms such as ‘electricity’, ‘shameful’, ‘government’, and ‘America’. The majority of these tweets coincided with the power outages in January, June, and July 2021, as well as the period following Trump's withdrawal from the Paris Agreement in June 2017. To gain deeper insights into the level of support for the Paris Agreement in Iran, a thematic analysis was conducted on a random sample of 500 tweets, selected based on the theoretical saturation of themes and sub-themes. The analysis revealed that 8 % of users expressed a positive orientation, 60 % a negative orientation, and 32 % a neutral orientation towards the Paris Agreement. The thematic analysis suggests that the negative sentiment is primarily due to frequent power outages, perceived inefficiency and lack of trust in government decisions, lack of transparency, and the perceived colonial nature of the agreement for developing countries. The findings underscore the importance of garnering public support in developing countries for international environmental agreements.

## 1 Introduction

The Paris Agreement is an international accord within the framework of the United Nations on climate change, which aims to address the issue of global climate change, adopted on December 12, 2015, at the 21st conference of parties to the United Nations Framework Convention on Climate Change in Paris; the Paris Agreement has been ratified by nearly 200 countries [1,2]. This agreement has been widely discussed by governments, organizations and individuals around the world [3]. In recent years, social media platforms such as X-previously known as Twitter-have become an important source of public opinion on various issues, including climate change [4].

Twitter is an effective platform for analyzing public opinion on the Paris Agreement due to its openness, real-time engagement, and diverse participation. It captures grassroots perspectives, highlights local-global connections, and amplifies voices often excluded from traditional media, providing researchers with valuable insights into societal attitudes and awareness of climate policies. The abundance of data generated by Twitter users creates opportunities for researchers and analysts to study public opinion on a wide range of topics, including politics and international relations, social and cultural trends, and health and environmental issues [5].

In Iran as well, the Paris Agreement has been a matter of concern for policymakers, researchers and the public as a controversial issue due to environmental and economic challenges [2]. The Paris Agreement is crucial for Iran as it addresses its severe environmental challenges. It offers a framework for adopting sustainable practices and reducing greenhouse gas emissions. As a major oil and gas producer, Iran's participation is vital for global climate goals and transitioning to a low-carbon economy. Economically, it could attract foreign investment and modernize Iran's energy sector, while politically, it enhances Iran's global standing. The agreement provides Iran an opportunity to balance economic growth, environmental sustainability, and international cooperation for long-term resilience.

Iran has signed the agreement but has not ratified it yet. This means that while Iran has indicated the intention to join, it has not been legally bound by its terms. Iran argues that it is difficult to implement the agreement due to severe international sanctions. Another reason is that there is some political opposition within Iran to joining the agreement in consideration of international conflict (specifically between Iran and the United States). Iranians' views on other environmental initiatives, such as the UN Sustainable Development Goals (SDGs) and the Kyoto Protocol, were relatedly influenced by economic challenges, political dynamics, and environmental concerns. The political framing of these initiatives, especially their perceived alignment with Western agendas, can lead to mistrust. Geopolitical tensions with Western nations further exacerbate this skepticism. However, when it comes to regional initiatives, there is increasing recognition in Iran of the need for environmental cooperation, particularly on shared issues like water management and air pollution, which could promote closer collaboration with neighbouring countries.

Like other countries, social media captures grassroots perspectives, reflecting how Iranians balance economic reliance on fossil fuels with the urgent need for sustainable development. These discussions often highlight the unique environmental challenges Iran faces, such as water scarcity and desertification, and connect them to broader climate action debates. They also reveal the aspirations of younger, tech-savvy generations and civil society in advocating for environmental policies.

By analyzing these conversations, policymakers and international organizations can better understand Iran's domestic priorities and challenges, enabling more effective engagement and fostering global awareness of the country's role in addressing climate change within its cultural and geopolitical context. In Persian Twitter, the analysis of public opinions on other topics such as health, environmental, social and cultural issues have been studied [[6], [7], [8], [9]].

Research on public perceptions of the Paris Agreement in developing nations, particularly Iran, remains limited. While social media platforms like Twitter are recognized for capturing public opinion on global issues, studies have predominantly focused on developed nations, with insufficient attention to Persian-language Twitter. Iran's unique context—marked by economic reliance on fossil fuels, severe environmental challenges, international sanctions, and political opposition—adds complexity to its public discourse on the agreement, which remains underexplored.

Existing studies on Persian Twitter have analyzed public opinion on various topics, but a comprehensive examination of climate policy discussions, especially regarding the Paris Agreement, is lacking. This study addresses these gaps by analyzing Iranian Twitter discourse, offering insights into public sentiment and the socio-political factors shaping perceptions of the Paris Agreement in a developing nation's context. To this end, the following research questions are addressed: (1) What is the Twitter users' level of support for the Paris Agreement? (2) What are the main topics of discussion among Twitter users about the Paris Agreement? (3) What events have provoked the most reactions to the Paris Agreement in Iran?

## 2 Literature review

Beyond the borders of Iran, several studies have investigated public opinion about climate change on Twitter by conducting sentiment analysis and topic modelling on a large corpus of data or thematic analysis on a subsample of big data [4,[10], [11], [12], [13], [14], [15], [16], [17], [18], [19], [20]]. Closer to the objectives of this paper, Müller-Hansen et al. [21] examined the German coal discussion on Paris climate mitigation objectives on Twitter before, during, and after the Coal Commission session for three years. They investigated whether and how the commission's work translated into shared perceptions and sentiments in the Twitter public discourse. They discovered that the sentiments of the German coal debate on Twitter grew increasingly negative over time. In addition, the sentiment became more polarized over time as both negative and positive language usage increased. These results suggested that the Coal Commission did not have a Twitter consensus regarding coal. While the debate on social media represents only a portion of the national debate, it provides policymakers with valuable information for evaluating public debates. However, few studies have investigated public opinion of twitter users regarding international environmental policies in developing countries, including Iran.

Nevertheless, several articles have analyzed Twitter users' opinions in various fields, such as Iran's political, historical, medical and environmental issues. Some Scholars have conducted sentiment analysis and topic modelling on many tweets to investigate public opinion on COVID-19 issues [9,22]. Some studies have investigated public opinion regarding online social activism in Iran, such as the study on the activism of Twitter users in commemorating the 33rd anniversary of the chemical bombing [23]. Majdizade and Molaei [8] *studied the diaspora or community far from the Iranian homeland.* Analysis of Iranian users' opinions about Iran-related policies has also been an interesting topic, such as the study of public opinions about the JCPOA by Abedin et al. [7].

Among research in the field of environmental issues, Ameli et al. [24] investigated how environmental journalists and activists active on Twitter frame the water crisis based on environmental justice. They monitored the Twitter pages of 30 environmental journalists and activists active on Twitter between January 1, 2017, and June 5, 2019, and collected tweets about the water crisis. The findings of the research showed that the most important components related to the framework of social justice in the field of water, from the point of view of environmental journalists, were: the lack of attention to the rights of ethnic groups or neglecting it in the issue of water, the dominance of the center-periphery model in water management plans and transferring water from the peripheral provinces to the central provinces, water and gender.

From the methodological point of view applied in this paper, several studies have investigated Twitter data from a qualitative perspective to gain deep insights into debates on Twitter by defining themes (e.g. Refs. [[25], [26], [27]]). Several papers also investigate the political dimensions of climate change discussions on Twitter through thematic analysis. Chen et al. [12] analyzed 5 million English tweets from 2018 to 2021. They discovered that climate movement actors on Twitter advocated for political actions and policy changes in addition to addressing climate-related social justice issues. Twitter conversations regarding the climate movement were intensely partisan, with many tweets directed at politicians, partisans, and country actors. In terms of raising awareness, the major themes in tweets included (1) attributing responsibility to various actors and targets such as politicians and industries, (2) highlighting climate consequences such as sea-level rise and rising temperature, and (3) raising awareness of the urgency of the issue. In terms of calling for action, the major themes included (1) calling for policy changes directed at resolving climate change, (2) highlighting action taken by youth activists, such as participating in the school strikes, convening at the UN Climate Summit, (3) calling for political action such as voting or elected certain candidates, (4) discussing the justice issues of climate strike such as pointing out how climate change might affect intersectionality issues in our society, and (5) offering climate solutions such as using wind and solar power. The findings of this study contributed to the comprehension of how individuals use social media to frame political issues and collective action.

Andreotta et al. offered their strategy to conduct a qualitative analysis of social media data by analyzing the topics of Australian Twitter commentary on climate change, using quantitative (non-negative matrix inter-joint factorization; topic alignment) and qualitative (thematic analysis) techniques. They identified four themes: (1) calls for action and increasing awareness, (2) discussions about the consequences of climate change, (3) policy debate about climate change and energy, and (4) local events associated with climate change. They offered useful insights for communicators and policymakers hoping to understand and engage the Australian online public [14].

## 3 Theoretical structure: The public sphere online

According to Habermas (1989), the public sphere consists of political discourse between the press, institutions of political discussion such as parliament, literary salons, and other public spaces. The public sphere was a space where ordinary people could congregate and deliberate without interference from the state or the church, and the most crucial aspect of Habermas' conception was that anyone could participate in this public sphere [28].

With the emergence of the internet and other new online media that can provide new communication spaces for dialogue, the debates surrounding the concept of the public sphere have taken on a renewed sense of importance. Although Habermas's original work was published long before the digital revolution, computer-mediated communication has replaced coffee house discourse [29]. It is said that the Internet generates new public spheres for political intervention, thereby expanding the arena for democratic participation [30].

Although several scholars have discussed the limitations of social media as well as its advantages in concerns such as manipulation of individuals and social control [[31], [32], [33], [34]], Social media platforms like Twitter have remained as an important public sphere in modern society. Even though the Twitter platform is restricted in Iran, it has remained an important public sphere and a platform for online activism [[6], [7], [8], [9]].

Hence it can be concluded that the theoretical contribution of this study lies in its application of Habermas's concept of the public sphere to the digital context of Twitter, particularly for climate change discourse in Iran. It reinterprets the public sphere as an online space where individuals, including marginalized voices, can engage in political and environmental discussions despite official restrictions on the platform. This adaptation highlights the resilience of online activism in constrained environments and positions Twitter as a modern public sphere facilitating democratic participation and collective action. The study bridges the gap between public sphere theory and environmental politics by analyzing how Twitter users frame, discuss, and mobilize around climate issues, linking global debates to Iran's unique socio-political and environmental challenges. Furthermore, it emphasizes Twitter's role as a platform for both political discourse and activism, reflecting localized expressions of global climate concerns. By situating Iranian Twitter users within the global climate conversation, this research expands the understanding of digital public spheres as tools for engaging with environmental policies, particularly in developing nations facing geopolitical and economic constraints.

## 4 Research method

26,411 tweets with Persian keywords having the same meaning as the Paris Agreement, were extracted from Twitter. After removing duplicate data, 25,851 tweets remained. To get more in-depth information about the themes of the Paris Agreement discussion on Twitter, a random sample of 500 tweets to be analyzed by the qualitative thematic analysis method (using Snscrape library in Python). In the analysis of themes, when needed, the link of the tweet in the main position on Twitter was studied more closely to better understand its meaning in relation to other tweets. Thematic analysis is a process for analyzing textual data that transforms scattered and diverse data into rich and detailed data. In other words, thematic analysis is conceptualizing and identifying central themes to discover what the data says [35]. It should be also noted that we do not consider English-language tweets on this topic. While English-language tweets may capture views of globally oriented or educated Iranians, they often lack connection to the broader population's realities. Additionally, incorporating English tweets risks overrepresenting elite perspectives and complicating analysis.

In this research, the sampling of tweets continued until theoretical saturation was reached. Theoretical saturation occurs when additional data do not help to complete and specify another theoretical category, and the samples appear similar [36]. Tweets were analyzed using the inductive method of thematic analysis, inspired by Braun & Clarke [37]. These steps included getting familiar with the data by reading a sample of tweets, creating primary codes and coding all the data in Excel, checking codes to identify themes in the entire data, reviewing and modifying themes, identifying and naming themes, and producing the report. The authors performed the primary analyses. After this initial analysis, two other people from other scientific fields reviewed and modified the codes and themes. The revised analysis was reviewed with several other experts, and appropriate corrections were made. The methods of increasing research accuracy include triangulation of analysts (as mentioned above) and a detailed description of the process [38]. In the end, the sample of tweets was re-examined, and the reliability between the evaluators in themes was between 92 % and 100 % and in sub-themes between 85 % and 100 %. The most frequent words in each theme were obtained using “wordcounter.net”.

## 5 Results

According to the results of the most frequent words in the data set other than the words with the same meaning as the Paris Agreement, the words ‘electricity’, ‘shameful’, ‘Iran’, ‘government’, ‘Trump’, ‘America’, ‘JCPOA’, ‘country’, ‘signature’ appear as the main topics in the word cloud.

**Fig. 1:**
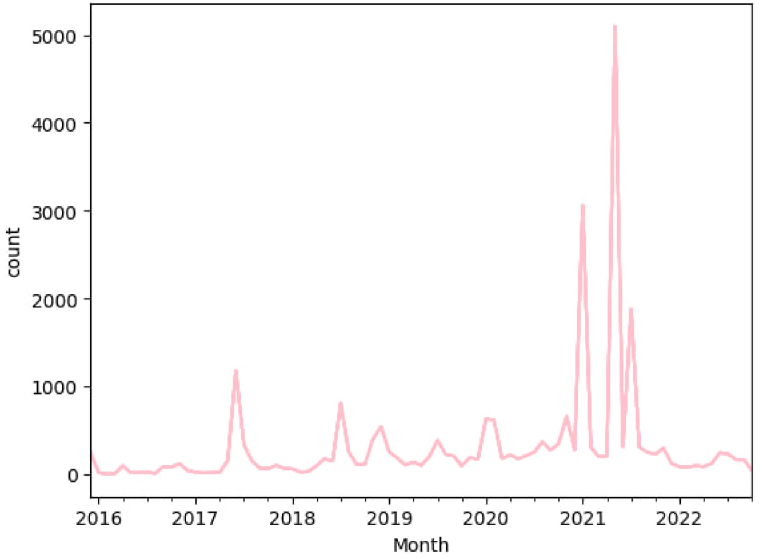
Frequency of tweets across time.

Examining the trend of tweets in Fig. 1 also shows that a significant number of tweets about the Paris Agreement were published in May, January and July 2021, respectively, according to the frequency. By examining these dates with Google Trends, we concluded that the frequent power outages on these dates and their connection with the implementation of the Paris Agreement in people's minds caused impulsive reactions among people. Also, on June 1, 2017, when Trump announced its withdrawal from the Paris Agreement, Persian Twitter users published a significant number of tweets related to the Paris Agreement. Therefore, Trump's withdrawal and power outages have caused the most reactions among Persian Twitter users about the Paris Agreement.

In the 500 tweets sample for thematic analysis, the words ‘Iran’, ‘the shameful Paris Agreement’, ‘electricity’, ‘America’, ‘JCPOA’, ‘Trump’, ‘government’, ‘country’, ‘foreign’, and ‘people’ were the most frequent.

In a thematic analysis of the 500 tweets, 50 ambiguous and unrelated data to the Paris Agreement were removed. Three main themes were obtained: positive orientation toward the Paris Agreement, negative orientation toward the Paris Agreement and neutral orientation toward the Paris Agreement. Themes, subthemes and some examples are illustrated in Table 1.

**Table 1:** Thematic coding of Persian tweets regarding the Paris Agreement.

Theme	Sub-theme	Translation
*Positive orientation toward the Paris Agreement (8 %)*	*Positive attitude toward the implementation of the Paris Agreement in Iran (3.7 %)*	*“Are our people in trouble now because of the implementation of the Paris Agreement or because of climate change, drought and waterlessness?! The people of Khuzestan will die in 60° due to warming in 30 years.”*
		*“Iran's type of commitment to the Paris Agreement is the so-called ‘No Regret Plan’. If we only halve the pollution of our cities, we have fulfilled our commitment.”*
	*Positive attitude toward the implementation of the Paris Agreement without mentioning Iran (4.3 %)*	*“Exactly, the Paris Agreement was formed based on all participating countries' contributions and the collective movement toward cleanup. At that time, there were the same numbers, and the agreement became possible only when each signatory country had a long-term plan to reduce pollution from its current point*.*”*
		*“Historic Paris Agreement saving the Earth”*
*Negative orientation toward the Paris Agreement (60 %)*	*Negative attitude toward the government (28.8 %)*	*“Expenses, problems and hardships for all the people without us … the plan and the secret agreement with us and without the people. #Paris_Agreement”*
		*“Mrs. Ebtekar, when you still don't know the difference between SO2 and CO2 gas, then you are making a mistake, signing the shameful #Paris_Agreement and causing billions of dollars damage to the country”*
	*Negative attitude toward the implementation of the Paris Agreement (10.8 %)*	*“The shameful Paris Agreement means damage to domestic production”*
		*“It was Trump who stood up against this mafia, that's why all the media were against Trump, folks like Mr. Darwish don't have proper political education and don't know what is behind the scenes of the Paris Agreement and they only see the appearance of the case”*
	*Orientation toward power outages (20.4 %)*	*“Friends, the electricity power is cut off and the internet is getting cut off little by little. I want to tweet you with fire and smoke with the hashtag.*
		*#shameful_Paris_Agreement”*
		*“The situation was the same last year and it could have been predicted that it will get worse. The best and fastest way is to set up gas power plants with a small capacity but to a large quantity, which this government has shut down because of the signing of the Paris Agreement …”*
		*“Maybe it's election time they are playing with us and want to show people a corner of hell. Otherwise, such incompetency is so strange. Even an elementary school kid knows how to plan #outrage, #shameful Paris Agreement.”*
*Neutral orientation toward the Paris Agreement (32 %)*	*Mentioning news related to the Paris Agreement (14.7 %)*	*“Zarif signed the Paris Climate Change Agreement.”*
	*Referring to events outside Iran regarding the Paris Agreement (7 %)*	*“Macron invited the American people, elites and entrepreneurs who are disappointed with the cancellation of the Paris Agreement to come to France … for the first time I wish I was an American”*
		*“G20 leaders criticize Trump for pulling out of Paris accord, but their own public budget for fossil fuel is still four times that of renewables*.*”*
	*Analysis of American leaders considering their decision regarding the Paris Agreement (5.8 %)*	*“Trump's America's opposition to internationalism had several important examples: withdrawing from the Paris Agreement, withdrawing from the JCPOA, withdrawing from the World Health Organization, withdrawing from the Human Rights Council, Biden's America has operationalized the return to these agreements except for the return to the JCPOA*.*”*
		*“How can you trust a president who easily abandons his country's agreements with the international community, such as the Paris Agreement, withdraws his signature from the G7 agreement on the plane, and suddenly enters into negotiations with North Korea, which threatened to annihilate him two months ago? How can we trust him?”*
	*Reacting to the arguments of opponents of the Paris Agreement without declaring a clear orientation on the Paris Agreement (4.5 %)*	*“No fool considers the Paris Agreement as the reason for the lack of electricity, because Iran is not committed to any treaty at all”*
		*“Secondly, this year's situation has nothing to do with the Paris Agreement, fuel and such things. The country's electricity and gas needs are increasing during a process for which a power plant should be built. The fact that the power plant, which should have been built two years ago, is not built is a problem now.”*
		*“Why are you linking everything to the #Paris_Agreement, which has not been implemented in Iran at all … ?!”*

### 5.1 Positive orientation toward the Paris Agreement

The tweets related to this theme supported the Paris Agreement (8 % of tweets). In general, this group of tweets stated their positive orientation in two ways: In some tweets, users expressed their positive attitude toward the implementation of the Paris Agreement in Iran. In some other tweets, users expressed their positive attitude toward the Paris Agreement without a specific orientation regarding its implementation in Iran. The words ‘Iran’, ‘Climatic’, ‘Earth’, ‘Trump’, and ‘World’ were the most frequent in tweets of this theme.

#### 5.1.1 Positive orientation toward the implementation of the Paris Agreement in Iran

In 16 tweets (3.7 % of tweets), users paying positive attention to Iran's situation in accepting this agreement pointed to: Iran's problems of drought and dehydration, opposition to extremists in dealing with the Paris Agreement, an invitation for the analysis of the Paris Agreement from political and scientific viewpoints, and Iran's lack of significant obligations in the case of joining the Paris Agreement, the carbon dioxide effects of Iran's petrochemicals, the benefits of its implementation such as the promotion of renewable energy projects, mentioning the agreement as a no-regret plan, and listing suggestions for its implementation in Iran. Some examples:

“Only a small petrochemical company emits 30 tons of carbon dioxide per hour. #paris_agreement”

“In the context of the #Total contract or the #Paris Agreement, two issues are important, leaving the political purpose and a sober scientific approach and sympathetic acceptance of criticism from the government, otherwise …”

#### 5.1.2 Positive orientation toward the implementation of the Paris Agreement without referring to the situation in Iran

Some tweets, regardless of the situation of Iran toward the Paris Agreement, have shown their positive attitude toward the Paris Agreement. They have pointed to factors such as the disastrous effects of Trump's withdrawal from the Paris Agreement, positive feedback toward Biden's return, criticism of the insufficient efforts of governments, saving the earth in the agreement, control of warming, dimensions of reaching sustainable development and collective movement of countries (21 tweets, 4.3 % of tweets).

“Economic powers and industrialized countries are not trying as they should to achieve the goals set in the Paris Climate Agreement. There is still the risk of the earth warming more and the possibility of warming up to …”

“Bravo! Minutes ago, in his first action, Biden officially returned to the Paris climate change agreement. In short, the course of actions that will continue to save the environment will officially begin, and maybe we can be saved from this disaster!”

### 5.2 Negative orientation toward the Paris Agreement

52 % of the tweets had negative attitudes toward the Paris Agreement. They have shown their negative attitude in three ways: negative attitude toward the actions of the government with the implementation of the Paris Agreement, negative orientation at the same time as the power outages in Iran and linking it to the Paris Agreement, and negative attitude toward the implementation of the Paris Agreement in Iran by mentioning some other reasons. Among the negative feedback, ‘shameful Paris Agreement’, ‘electricity’, ‘government’, ‘country’, ‘power outrage’, ‘production’, ‘Ebtekar’, ‘people’, ‘signature’, and ‘JCPOA’ are the most frequent tweets.

#### 5.2.1 Negative orientation toward the government

The government in this context means the executive branch, including the presidency and Council of Guardian (constitutional council) and the legislative branch or the Islamic Consultative Assembly (Iranian parliament) in Iran. A significant number of tweets (28.8 %) have shown opposition to the Paris Agreement in connection with the government's negative actions.

In particular, they have severely criticized the performance of the head of the Environmental Organization (Masoume Ebtekar), the President (Hassan Rouhani) and the Minister of Foreign Affairs (Mohammed Javad Zarif). They have called these people Westernized, illiterate and traitors due to the approval of the Paris Agreement. They have also attributed the implementation of this agreement to the personal interests of politicians.

“Inflation, stagnation, unemployment, injustice, poverty, aristocracy, turning back and kneeling in front of enemies, false promises and imprudence, reducing the country's power, and the controversial and humiliating agreements of JCPOA, 2030, FATF, IPC, Paris Agreement and … It was the result of westernized and reformist responsibility.”

“Mrs. Ebtekar! How many victims did your family interest initiative call for in the Paris Agreement?”

While complaining about the politicians, some have demanded the punishment and trial of the decision-makers for the Paris Agreement's approval in Iran.

“God bless you in this world and the hereafter. God willing, we will leave the Paris agreement completely, and complain to Mrs. Ebtekar about the damages this accord has caused the country.”

*“Masoume Ebtekar should be punished for signing the* shameful *Paris Accord!”*

Criticizing the signing of the Paris Agreement without preconditions and the government's secrecy in approving the Paris Agreement, some users have asked for transparency from the government.

“Environmental Organization officials should clearly report the amount of obligations and (according to Masoumeh Ebtekar) the advantages of the #Paris_Agreement to the representatives, not like the JCPOA with a wrong translation within 20 minutes. The people you have voted for a representative, be smart!”

Some users believed that raising issues such as cycling and the right of women to be present in the stadium was aimed at diverting the flow of public opinion from the Paris Agreement signing.

“@ebtekarm_ir, when you reach an impasse, you look for these things to distract us: #stadium or #bicycle or #right, but now people are focused: #national_money_value #Paris_Agreement”

Some users have addressed the structural problems of the ratification process of the Paris Agreement. This agreement was approved without approval from the Islamic Council or the final approval of the Guardian Council.

“Isn’t it true that the Paris Agreement has not been approved by the Council of Guardians? So how can the government implement it? Before criticizing the government, criticize the country's failed laws that allow the government to circumvent the law. The incompetent parliament must prevent this by ratifying laws.”

“It is said that the #electricity_cut off is related to the #Paris_Agreement. There is no hope for the government, but #parliament cannot examine this accord and give a report to the people??@mb_ghalibaf”

“Every day the news comes out about secretly joining a dangerous international #convention. According to the constitution, all international treaties must be approved by the parliament. Where exactly is the parliament in the affairs?! Why has this parliament become so inefficacious?!# paris_agreement, #Larigani_lobby, #interaction_with_wolves.”

In online activism, there is usually a central icon that forms the core of activist discussions. In the discussion of the Paris Agreement on Twitter, a significant number of users have shown their dissatisfaction with the implementation of the Paris Agreement with the hashtag “(ننگین) ‘shameful’ Paris Agreement”, which indicates their regret over the implementation of this agreement. This word is used in 20 % of all tweets in the sample.

“How interesting … last night, the subtitled news channel showed that the cause of the outage was high electricity consumption and cooling devices, which means the nation is to blame. How rudely they say something every day. It sounds like the shameful Paris Agreement hashtag has become hot.”

#### 5.2.2 Negative orientation by mentioning the disadvantages of the implementation of the Paris Agreement

A significant number of users (10.8 % of tweets) have shown a negative attitude toward the implementation of the Paris Agreement due to several reasons. Problems such as the reduction of the country's economic growth as a result of the implementation of the Paris Agreement, the cost of international agreements, the creation of problems for the export of electricity in Iran, the loss of the opportunity to develop crypto-currency technology due to the significant consumption of electricity of miners, and the non-use of raw resources due to the implementation of the Paris Agreement in Iran.

“The victory of the #resistance_front to crush the #Isis sedition and Iran's role in realizing this victory should not make us forget the #Paris_agreement and its heavy economic and security obligations of 50 billion dollars.”

“Tell a ten-year-old child, I'll give you a bag of chocolates, it's all yours, but you don't have the right to touch it, he'll take it and hit you. Now we have the most resources and … #shameful-Paris_Agreement”

A significant number of users have a conspiracy theory view regarding this agreement and have listed the colonial aspects behind the scenes of these types of agreements.

“The Paris Agreement is to increase productivity and reduce air pollution as a nice-looking affair. However, it uses industrial terrorism against less developed countries and hinders their progress. The Westerners ruined the air during the industrialization period and now they are worried about the rest!”

“All the international agreements and contracts are for the control of the countries by the Westerners so that they can dominate the world more. The JCPOA, the Paris Agreement, FATF, etc. are all agreements that limit the power of independent countries”

Some users have also pointed to the issue that Iran accounts for a small share of pollution emissions, or they have stated that Iran will not be able to receive international aid due to international sanctions.

“Have you ever wondered why many rich oil countries (Middle Eastern countries) refused to accept the Paris Agreement? The answer is clear, because of the financial loss of this accord for countries with oil resources! Is it correct that a sanctioned country causes financial loss to itself? #The shameful Paris Agreement”

Some users have considered Iran's stay in the Paris Agreement illogical since Trump withdrew from the accord even though America could have contributed much more to the pollution reduction. In comparison, the role of Iran could be easily ignored.

“America is the biggest destroyer of the world's environment and officially produces 25% of the world's greenhouse gases! P.S.: Now America will withdraw from the Paris Agreement rudely!! Then some traitors threw the country into the Paris Agreement and stopped any progress …”

Some users basically denied the issue of climate change. In the meantime, a film by Ali Akbar Raefipour (an Iranian political celebrity) has received much attention, which has explained the deception of climate change caused by human activities.

“Wasn’t it claimed that the planet has warmed up? Freezing in America and snow in Arabia!!! Where are those who believe in the #ParisAgreement in the parliament? Professor Raefipour's lecture, “Scrutinizing the #Paris_Agreement”, spend about 3 hours to see what a scandal it is. #electricity_outage”

#### 5.2.3 Orientation to power outages

All tweets that contain comments about power outages and their relevance to the Paris Agreement are included in this sub-theme. This subtheme also includes tweets that question the government's performance or explain the reason for opposing the Paris Agreement in the case of power outages, and these types of tweets are not included in other subthemes.

As shown in the trend of the number of tweets over time, in the dates of January, June, and July 2021, frequent power outages led to the emergence of a significant number of tweets that related the power outage to the implementation of the Paris Agreement (20.4 % of tweets). The discussion of the power outage has been one of the most important factors in the activism of users and the discussion of the Paris Agreement on Persian Twitter.

“The JCPOA, the Paris Agreement, which obliges us to reduce the greenhouse gas emissions of industrialized countries, the discussion of issuing the necessary permits, and the imprudence of the officials have caused an increase in electricity consumption and a decrease in electricity production this year, and this unbalanced process has caused many problems for people, producers and industries.”

“May God curse the traitors who signed the Paris Agreement to make such a nation fall into hardship in this heat …”

“Successful management means you do something that instead of being upset about the power outage, people are upset about why you didn't cut it off on schedule and you didn't know about it before #shameful_Paris_Agreement”

### 5.3 Neutral attitude toward the Paris Agreement

32 % of all tweets, despite discussing the Paris Agreement, did not have an explicit personal orientation toward supporting or not supporting the Paris Agreement. These tweets published news related to the Paris Agreement, discussed the issues related to the Paris Agreement abroad, analyzed the American leaders considering their decision regarding the Paris Agreement, or reacted to the arguments of opponents of the Paris Agreement. Among tweets with a neutral attitude, the words ‘America’, ‘Trump’, ‘withdrawal’, ‘JCPOA’, ‘Agreement’, ‘Climatic’, ‘Water’, ‘Iran’, and ‘China’ are the most frequent tweets.

#### 5.3.1 Mentioning news related to the Paris Agreement

Some tweets spread the news about the Paris Agreement (14.7 % of tweets): news related to the performance of world leaders in the context of the Paris Agreement, countries' actions, analysis of the Paris Agreement, and republishing politicians' statements about the Paris Agreement.

“The new research of Science magazine (#Elam) says that even if the efforts meet the #Paris_Agreement and the #warming_of the earth is limited to the threshold of 2 °C, #crops will still suffer from the expansion of #insects.”

“Disruption of Canadian Prime Minister's speeches and the rise of #yellowvests in Canada: opposition to globalization, mass immigration, special attention to minorities and environmental policies of the Paris Agreement”

“Trump: I withdrew from the Paris Agreement because it was very unfair. We have the best weather without hurting American industry.”

#### 5.3.2 Referring to events outside Iran regarding the Paris Agreement

Some users have analyzed the events related to the Paris Agreement abroad in their tweets without specifying their attitude toward the Paris Agreement (7 % of tweets).

“In my opinion, the biggest challenge of #Trump's withdrawal from the #Paris Agreement is for Hollywood and the credibility of its films, which often present America as the saviour of humanity and the planet.”

“Withdrawal from the #Paris Agreement, withdrawal from #UNESCO and now #JCPOA? It will lead to an unprecedented decrease in the international credibility of America.”

#### 5.3.3 Analysis of American leaders considering their decision about the Paris Agreement

American decisions regarding the Paris Agreement, such as Trump's withdrawal from the Paris Agreement in June 2017 and Biden's return to the Paris Agreement on the first day of his presidency on January 20, 2021, have created feedback from Persian Twitter users about the analysis of these types of decisions and the discussions around them (0.8 % of tweets). Discussions include the analysis of Trump and Biden's personalities based on their decisions regarding the Paris Agreement, Biden's return to the Paris Agreement and not returning to the JCPOA, and the analysis of America's behaviour toward policies, intensifying mistrust of America.

“(This is the method of those who) do not observe kinship and covenant regarding any believer. And they are the rapists!

(Quran: Tobe/10)

#JCPOA

#Paris_Agreement”

“Biden was absolutely right, the Democrats solved the terrible recession of George Bush's time and handed it over to Trump, most of Trump's words are lies, and he messed up the world order. Under the pretext of patriotism, he messed up the world economy, withdrew from all his commitments, NAFTA, The Paris Agreement and …, the difference between a politician and a populist person is clear here.”

#### 5.3.4 Reacting to the arguments of opponents of the Paris Agreement without declaring a clear orientation toward the Paris Agreement

Some tweets have raised issues in response to opponents' arguments about the Paris Agreement without clearly stating their orientation toward it (14.7 %). For example, some Twitter users have pointed out that the recent power outages have nothing to do with the Paris Agreement and have attributed the power outages to factors such as weak management and miners’ power usage. Some believed that the spread of the idea of attributing the power outage to the Paris Agreement is a media game, and the main reason is the spread of using miners to extract cryptocurrency.

“Hello, it has nothing to do with the Paris Agreement. The amount of industrialization or production of greenhouse gases in Iran is not numerical and we are not comparable with other countries, and if we shut down the entire country, we will not have a significant effect because we have no industry.”

“It seems that the production of bitcoins by the Chinese is your red flag, and why don't you say that around 4.5% of the world's bitcoins with subsidized electricity (a very free dollar price) should be produced in Iran? Like producing watermelons in the low-water country of Iran for export. And the order has come, forget everything, attack the Paris Agreement.”

The results reveal that discussions about the Paris Agreement in Iran are highly political, often focusing on government shortcomings and attributing blame to the United States. This political framing suggests a need for reforms to address public concerns, justify economic challenges, and build trust in international agreements, ensuring they are seen as beneficial rather than detrimental to developing countries.

Iranians' reluctance to support the Paris Agreement largely stems from prioritizing economic challenges over environmental concerns. Developing countries often view climate commitments as potential obstacles to economic growth, in contrast to developed nations where environmental issues may take precedence [39]. The perceived historical unfairness of the Paris Agreement also plays a role. Some view the agreement as unjust because developed countries have historically contributed more to emissions. This finding aligns with which notes that public support in developing countries is influenced by perceptions of fairness and the distribution of climate action costs [40]. This perception highlights the importance of addressing the differentiation of responsibilities under the Paris Agreement, where developing nations advocate for a fair distribution of obligations based on historical emissions and current capabilities, contrasting with developed countries’ focus on collective action without as much emphasis on equity [41,42].

Since mistrust in government plays a significant role in negative public sentiments, to enhance public trust, policymakers should be transparent about their positions on international environmental agreements and consider public opinions more seriously. Addressing concerns about the agreement's perceived colonial aspects is also crucial. Engaging in open dialogue about the benefits and drawbacks of such agreements with experts and the public can help alleviate these concerns.

Research by Maliniak et al. [43] shows that exposure to expert opinions positively correlates with public support for international climate cooperation, highlighting the need for informed public discourse. Supporting mediatory actors such as government bodies, NGOs, and environmental activists can improve information dissemination on social networks. Developing regulations to provide safe NGO activism and expert contributions is important.

## 6 Discussion

The findings of this study provide valuable insights into Iranian public sentiment towards the Paris Agreement, as expressed on Persian Twitter. The results reveal a nuanced landscape of public opinion, marked by significant political, economic, and social influences that shape perceptions of climate policy in Iran. This discussion situates the findings within broader global and national contexts.

### 6.1 Positive sentiment: A minority perspective

The minority of tweets expressing a positive orientation towards the Paris Agreement underscores limited but meaningful support for international climate commitments. Positive sentiments often emphasize the potential benefits of implementing the Paris Agreement, such as promoting renewable energy and addressing Iran's environmental challenges, including drought and air pollution. These tweets reflect an awareness of the global urgency of climate action and the interconnectedness of environmental issues.

However, the lack of specific focus on Iran's implementation in many positive tweets highlights a gap in localized advocacy and policy communication. To build on this support, policymakers and environmental advocates should prioritize public education and engagement, emphasizing how adhering to the Paris Agreement can address Iran's unique environmental challenges while fostering economic opportunities, such as green energy investments.

### 6.2 Negative sentiment: Dominated by distrust and economic concerns

The dominance of negative sentiment (52 % of tweets) reveals significant public skepticism towards the Paris Agreement. This skepticism is rooted in three primary factors:

A Mistrust in Government: Criticisms of government officials for perceived secrecy, incompetence, and self-serving motivations highlight a mistrust in government. This mistrust extends to the agreement itself, as many perceive it as a vehicle for advancing political elites' agendas rather than addressing national interests. The prevalence of the hashtag “shameful Paris Agreement” reflects a deeply entrenched sense of betrayal.
B Economic Concerns: Many users view the Paris Agreement as a threat to Iran's economic growth, citing its potential to hinder fossil fuel exports and industrial development. These concerns align with broader apprehensions in developing countries, where economic priorities often outweigh environmental ones. Additionally, the perception of the agreement as a “colonial” imposition exacerbates resistance, reflecting a historical mistrust of international frameworks perceived as favouring developed nations.
C Event-Driven Activism: The surge in negative sentiment during power outages indicates the role of immediate crises in shaping public discourse. Associating these outages with the Paris Agreement, whether factually accurate or not, underscores the importance of addressing misinformation and contextualizing environmental policies within broader infrastructural and governance challenges.

### 6.3 Neutral sentiment: A need for clarity and engagement

The neutral stance (32 % of tweets) reflects a lack of explicit alignment with or against the Paris Agreement. These tweets primarily focus on disseminating news, analyzing global developments, and responding to opponents’ arguments. This neutrality suggests a potential opportunity for engagement, as these users may be open to persuasion through informed discourse and transparent communication.

**Fig. 2:**
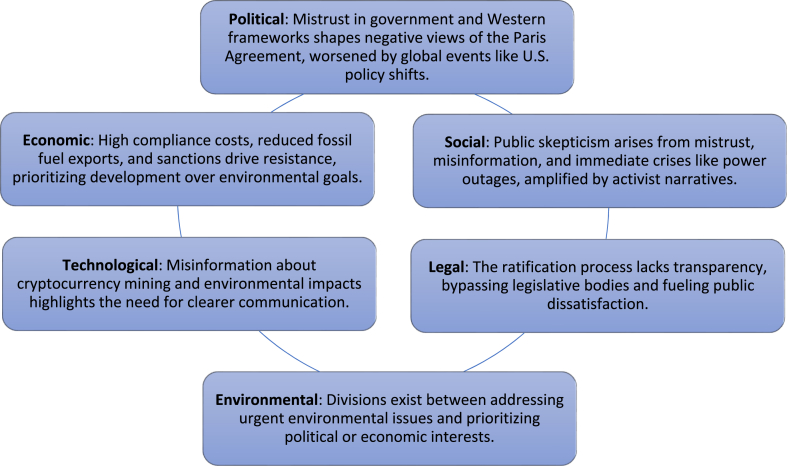
The summary of PESTLE analysis.

In addition to the above discussion, The PESTLE^1^ analysis may highlight key elements shaping sentiment toward the Paris Agreement in Iran from another perspective depicted in Fig. 2.

## 7 Conclusion

This study highlights the challenges and opportunities in fostering public support for climate agreements in developing countries. The findings highlight the importance of context-specific approaches to climate advocacy that account for political, economic, and historical factors unique to each nation. Building public trust, promoting equity, and ensuring transparent communication are essential for advancing global climate cooperation and addressing the critical environmental challenges of our time. The analysis underscores the significance of Twitter as a platform for virtual activism and public opinion formation in Iran. The dynamic and event-driven nature of discussions, particularly during times of power outages and international decisions like Trump's withdrawal from the Paris Agreement, highlights the interplay between domestic challenges and global developments in shaping public perceptions.

To address public concerns and build support for international environmental agreements like the Paris Agreement, the following policy implications are recommended:

A Enhancing Transparency: Policymakers should communicate openly about the agreement's provisions, implementation plans, and anticipated impacts, addressing concerns about secrecy and potential disadvantages.
B Strengthening Public Trust: Engaging civil society, fostering expert-led discussions, and providing platforms for diverse stakeholders can help rebuild trust and promote informed dialogue.
C Combating Misinformation: Educational campaigns and collaborations with trusted organizations can dispel misconceptions, particularly those linking the Paris Agreement to economic challenges like power outages.

The main limitation of this study is that distinctions between tweets from ordinary citizens, politicians, NGOs, and news agencies were not made, which could obscure variations in perspectives. Future research should address this limitation and explore the opinions of diverse societal groups, including policymakers, NGOs, and ordinary citizens. Investigating differences in sentiment across demographic factors, such as political ideology and gender, could provide deeper insights into the drivers of public opinion. Additionally, examining potential bot activity in climate-related activism could shed light on the dynamics of digital discourse.

## CRediT authorship contribution statement

**Faeze Atefinia:** Writing – original draft, Visualization, Software, Project administration, Investigation, Formal analysis, Data curation. **Seyed Reza Mirnezami:** Writing – original draft, Supervision, Software, Methodology, Investigation, Conceptualization.

## Data availability statement

Data used in this paper for qualitative analysis is available as supplementary material in the article submission.

## Declaration of generative AI and AI-assisted technologies in the writing process

During the preparation of this work, the authors used ChatGPT to paraphrase and decrease word count. After using this tool/service, the author) reviewed and edited the content as needed and took full responsibility for the content of the publication.

## Declaration of competing interest

The authors declare that they have no known competing financial interests or personal relationships that could have appeared to influence the work reported in this paper.

## Footnotes

1 PESTLE is an abbreviation for Political, Economic, Social, Technological, Legal, and Environmental analysis.
